#  Antimicrobial Drug Resistance of *Vibrio cholerae*, Democratic Republic of the Congo

**DOI:** 10.3201/eid2105.141233

**Published:** 2015-05

**Authors:** Berthe Miwanda, Sandra Moore, Jean-Jacques Muyembe, Georges Nguefack-Tsague, Ickel Kakongo Kabangwa, Daniel Yassa Ndjakani, Ankur Mutreja, Nicholas Thomson, Helene Thefenne, Eric Garnotel, Gaston Tshapenda, Denis Kandolo Kakongo, Guy Kalambayi, Renaud Piarroux

**Affiliations:** Ministry of Public Health, Kinshasa, Democratic Republic of the Congo (B. Miwanda, J.-J. Muyembe, G. Tshapenda);; Aix-Marseille University, Marseille, France (S. Moore, R. Piarroux);; University of Kinshasa, Kinshasa (J.-J. Muyembe);; University of Yaoundé I, Yaoundé, Cameroon (G. Nguefack-Tsague);; Ministry of Higher Education and University, Kinshasa (I.K. Kabangwa, D.K. Kakongo);; Field Epidemiology Laboratory Training Program, Kinshasa (D.Y. Ndjakani);; Wellcome Trust Sanger Institute, Cambridge, UK (A. Mutreja, N. Thomson);; London School of Hygiene and Tropical Medicine, London, UK (N. Thomson);; Hôpital d’Instruction des Armées Laveran, Marseille, France (H. Thefenne, E. Garnotel);; World Health Organization, Ouagadougou, Burkina Faso (D.K. Kakongo);; World Health Organization, Kinshasa (G. Kalambayi)

**Keywords:** antimicrobial resistance, bacteria, cholera, Vibrio cholerae O1, MLVA, whole-genome sequencing, El Tor variant, clone, Democratic Republic of the Congo

## Abstract

We analyzed 1,093 *Vibrio cholerae* isolates from the Democratic Republic of the Congo during 1997–2012 and found increasing antimicrobial drug resistance over time. Our study also demonstrated that the 2011–2012 epidemic was caused by an El Tor variant clonal complex with a single antimicrobial drug susceptibility profile.

Cholera is an acute intestinal infection caused by *Vibrio cholerae* (*1*). Although hydration remains the primary treatment for cholera, antimicrobial drug therapy is recommended for severely ill patients (*2*). However, multidrug-resistant *V. cholerae* strains have long been observed in Africa (*3*), and strains exhibiting new resistance phenotypes have emerged during recent epidemics (*4*). It is therefore critical to carefully monitor changes in strains’ susceptibility to antimicrobial drugs in each African country and adapt treatment recommendations accordingly.

Few longitudinal studies assessing shifts in the resistance of *V. cholerae* to antimicrobial drugs in Africa have been established. The available studies are limited either to a restricted area (*5*) or a short time period (*6*). We describe the long-term evolution of antimicrobial drug susceptibility of an extensive set of *V. cholerae* isolates collected in the Democratic Republic of the Congo (DRC). We applied whole-genome sequencing and multiple locus variable-number tandem-repeat analysis (MLVA) to clarify the mechanisms behind the aggressive epidemic of 2011–2012 that spread throughout the country, affecting regions to which cholera was not endemic (*7*).

## The Study

Sample collection included all available isolates from major outbreaks in the DRC during 1997–2012, which were stored at the National Institute of Biomedical Research. The Table shows the locations where the 1,093 tested isolates were collected.

**Table:** Distribution of 1,093 *Vibrio cholerae* isolates, by year and province, Democratic Republic of the Congo, 1997–2012

Year	Province, no. isolates										Total
	Bandundu	Bas-Congo	Equateur	Kasai-Oriental	Katanga	Kinshasa	Maniema	North Kivu	Oriental	South Kivu	
1997	0	4	0	0	0	2	0	0	0	0	6
1998	0	2	0	0	15	14	0	4	7	0	42
1999	8	0	0	0	1	22	0	0	0	0	31
2000	3	0	10	0	0	13	0	0	0	0	26
2001	0	0	0	0	16	0	0	0	0	0	16
2002	0	0	0	0	44	0	0	0	0	0	44
2003	0	0	0	19	21	4	0	0	0	0	44
2004	0	0	0	0	1	2	0	3	0	0	6
2005	0	0	0	0	1	0	0	2	0	0	3
2006	2	3	0	0	9	0	0	0	0	0	14
2007	0	0	0	0	15	1	0	1	5	0	22
2008	0	0	0	0	30	0	5	4	17	0	56
2009	0	0	0	0	112	0	0	2	0	11	125
2010	0	0	0	0	10	0	0	0	0	5	15
2011	46	0	17	0	0	73	4	151	45	0	336
2012	11	25	16	0	1	27	0	196	31	0	307
Total	70	34	43	19	276	158	9	363	105	16	1,093

*V. cholerae* O1 strains stored in nutrient agar during 1997–2012 were cultured on thiosulfate citrate bile salts agar and nutrient agar at the National Laboratory, Kinshasa, DRC. The strains were enriched in alkaline peptone water liquid medium and incubated at 37°C for 18–24 h. Biochemical and serogroup characterization was subsequently performed according to standard protocols (*8*).

Antimicrobial drug susceptibility testing of 1,093 confirmed *V. cholerae* isolates was performed by using the Kirby-Bauer disk diffusion method (*9*) with Mueller agar (bioMérieux, Marcy l’Etoile, France). *V. cholerae* O1 Ogawa and Inaba reference strains (ATCC) served as controls. Isolates were tested against 9 antimicrobial drugs as follows: ampicillin (10 µg), chloramphenicol (30 µg), sulfamethoxazole/trimethoprim (1.25 + 23.75 µg), tetracycline (30 µg), doxycycline (30 µg), norfloxacin (5 µg), ciprofloxacin (5 µg), nalidixic acid (30 µg), and erythromycin (30 µg) (bioMérieux). Interpretation of inhibition diameters (sensitive, intermediate, and resistant) was performed according to Clinical and Laboratory Standards Institute guidelines (*10*). When no interpretive criteria for *V. cholerae* were available from these guidelines, breakpoints for *Enterobacteriaceae* were applied by using *Escherichia coli* ATCC 25922 for quality control. The few intermediate results were categorized as resistant for this study. The strains were then regrouped into 21 resistance profiles. 

Seventy-four clinical isolates from the 2011–2012 epidemic, spatiotemporally representative of outbreak diffusion, were subcultured and transported to Marseille, France. In Marseille, the strains were recultivated and identified as previously described (*11*). For DNA extraction, a 50-colony aliquot of cultured cells was suspended in 500 µL NucliSENS easyMAG lysis buffer (bioMérieux). DNA was extracted by using a NucliSENS easyMAG platform (bioMérieux) according to the manufacturer’s instructions. MLVA-based genotyping of the *V. cholerae* isolates and eBURST analysis (http://eBURST.mlst.net) were performed as previously described (*11*). To perform a phylogenetic assessment of the core *V. cholerae* genome on the basis of genome-wide single nucleotide polymorphisms, whole-genome sequencing was performed on an isolate (L286) collected at epidemic onset by using a HiSeq Illumina System (Illumina, San Diego, CA, USA) as previously described (*12*).

**Figure 1:**
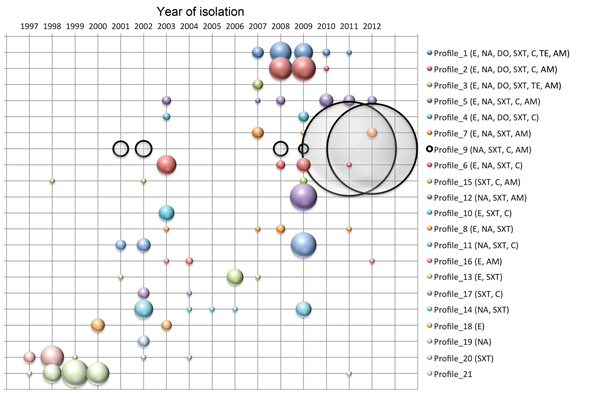
*Vibrio cholerae* strain antimicrobial drug resistance profiles plotted by year, Democratic Republic of the Congo, 1997–2012. On the basis of the antibiogram results, strains were grouped into 21 antimicrobial drug resistance profiles. The antimicrobial drugs to which the strains displayed resistance are indicated on the right. Circle circumference represents the relative number of isolates per profile. AM, ampicillin; C, chloramphenicol; SXT, sulfamethoxazole/trimethoprim; TE, tetracycline; DO, doxycycline; NOR, norfloxacin; CI, ciprofloxacin; NA, nalidixic acid; E, erythromycin.

**Figure 2:**
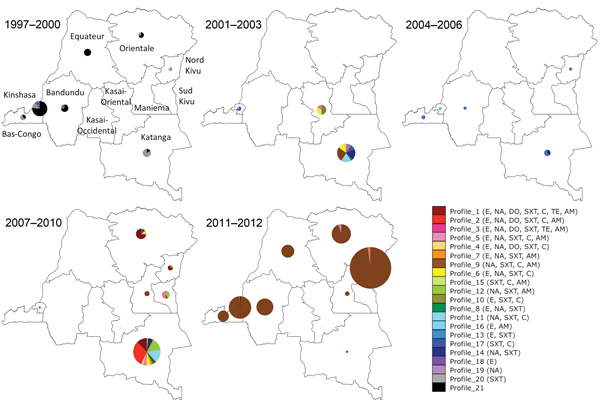
Spatiotemporal localization of isolate antimicrobial drug resistance profiles by time period and province, Democratic Republic of the Congo, 1997–2012. Strains were grouped into 21 antimicrobial drug resistance profiles. The antimicrobial drugs for which the strains displayed resistance are indicated in the lower right panel. Patterns of antimicrobial drug resistance were further grouped into 5 periods. Circle circumference represents the relative number of strains, while the colors correspond to the different antimicrobial drug resistance profiles. Provinces are indicated in the 1997–2000 map. The maps were generated by using QGIS version 2.4.0-Chugiak (http://qgis.org/api/2.4/). AM, ampicillin; C, chloramphenicol; SXT, sulfamethoxazole/trimethoprim; TE, tetracycline; DO, doxycycline; NOR, norfloxacin; CI, ciprofloxacin; NA, nalidixic acid; E, erythromycin.

A spatiotemporal analysis was performed on the basis of the antibiogram profiles of *V. cholerae* isolates collected in the DRC during 1997–2012. The strain profiles were plotted by year (Figure 1) and then mapped by year and province. Using these data, we regrouped them into 5 representative periods (Figure 2). The *V. cholerae* strains displayed an increasingly complex resistance phenotype to various antimicrobial drugs. Sulfamethoxazole/trimethoprim resistance was observed initially, followed by resistance to nalidixic acid, erythromycin, and chloramphenicol during the early 2000s. Although sensitivity to fluoroquinolones seemed to be preserved, strain resistance patterns continued to evolve with the circulation of isolates resistant to tetracyclines and ampicillin from 2007–2010. Finally, isolates collected during 2011–2012, which was marked by the westward spread of a major epidemic (*7*), displayed a single antimicrobial drug susceptibility profile: resistance to most antimicrobial drugs except cyclines and fluoroquinolones.

Serotype analysis of the 1,093 *V. cholerae* isolates showed that Inaba strains were restricted to the western region of the country, Ogawa strains were isolated in the east and south, and Hikojima strains were restricted to Oriental Province, in the northeastern region of the country. During 2001–2010, Inaba and Ogawa serotypes were observed, but Ogawa predominated; during 2011–2012, these serotypes switched, and the Ogawa serotype was almost completely replaced by Inaba.

To examine the particular 2011–2012 epidemic that spread throughout the DRC (*7*), 74 *V. cholerae* isolates were assessed by using MLVA and eBURST analysis. Overall, the isolates displayed 19 different MLVA genotypes, of which 18 grouped into 1 clonal complex. eBURST analysis indicated that the clonal complex likely arose from a founder strain identified at the beginning of the epidemic. Furthermore, whole-genome sequence analysis of an isolate identified in March 2011 in Lubunga, Oriental Province (L286), revealed that the strain was an El Tor variant with CTX-3 type phage and a RS1 satellite phage. Phylogeny analysis situated this DRC strain close to the major Kenyan clade in the most recent wave of the seventh pandemic (data not shown).

## Conclusions

Analysis of a panel of *V. cholerae* clinical isolates from the DRC from 1997–2012 highlighted a loss of sensitivity to leading antimicrobial drugs, although strains remain susceptible to fluoroquinolones. However, a risk for emergence and spread of fluoroquinolone-resistant strains exists, as has been shown elsewhere in Africa (*13*). Because resistance to nalidixic acid is frequently associated with decreased susceptibility to fluoroquinolones, nalidixic acid resistance must be detected to monitor the emergence of highly resistant strains (*14*).

Our findings also provide new insight regarding the cholera epidemic of 2011–2012. This epidemic appears to have been caused by the expansion of a specific *V. cholerae* subpopulation, which rapidly diffused countrywide. Furthermore, sequence analysis showed that the clone responsible for this epidemic, an El Tor variant with CTX-3 type phage, falls close to the major Kenyan clade in wave 3 of the seventh pandemic. This observation correlates with a 2011 study demonstrating that the seventh cholera pandemic had been caused by specific strains originating from a unique ancestral clone that have spread globally in successive waves (*12*). The 2011–2012 isolates displayed a specific antimicrobial drug resistance pattern, characterized by the return of tetracycline and doxycycline sensitivity. The outbreak strain also represented a serotype switch from Ogawa to Inaba. However, further MLVA genotyping of preoutbreak isolates is required to determine whether these strains were already present in the region or if they represent a new *V. cholerae* population.

This study demonstrates that molecular and microbiological analyses of *V. cholerae* isolates provide extensive insight into the mechanisms of cholera epidemics. MLVA and whole-genome sequencing are powerful tools for elucidating epidemic dynamics because these methods have been used to link distinct outbreaks and identify the origin of certain epidemic *V. cholerae* strains (*15*). Improved sampling of clinical isolates is essential to monitor changes in pathogen antimicrobial drug resistance and elucidate the dissemination pathways of toxigenic strains to ensure proper management of patients requiring antimicrobial drug treatment and to appropriately direct the public health response.
